# Emotional design study of sustainable denim clothing based on SEM: a case study of generation Z consumers

**DOI:** 10.3389/fpsyg.2026.1679815

**Published:** 2026-05-21

**Authors:** Jiayi Wang, Liyan Zhao, Shuguang Han

**Affiliations:** School of Fashion Design and Engineering, Zhejiang Sci-Tech University, Hangzhou, China

**Keywords:** denim clothing, generation Z consumers, Kansei engineering, structural equation model, sustainable design

## Abstract

**Introduction:**

Against the backdrop of global sustainable development goals, the green transformation of the textile and apparel industry is increasingly imperative. Integrating the Stimulus-Organism-Response (S-O-R) framework with Kansei Engineering theory, this study investigates the mechanisms through which design attributes associate the Purchase Decisions of Generation Z consumers within the sustainable denim market.

**Methods:**

Utilizing a cross-sectional convenience sampling approach, survey data were obtained from 432 Generation Z consumers born between 1995 and 2009. To test the 17 proposed hypotheses, Structural Equation Modeling (SEM) and Bootstrap mediation analysis with 5,000 samples were utilized.

**Results:**

The structural model demonstrated excellent fit, indicated by indices such as a CMIN/DF of 1.668, an RMSEA of 0.039, and a CFI of 0.983. The results reveal that Appearance Characteristics, Sustainability Values, and Emotional Values exert significant positive effects on Purchase Decisions. Thirteen of the 17 hypotheses were supported, showing that Sustainability Values functions as a central mediating variable linking product attributes including Appearance Characteristics, Functional Characteristics, and Symbolic Characteristics to Purchase Decisions. However, the mediating role of Emotional Values is contingent upon specific attributes: it significantly mediates the relationship between Appearance Characteristics and Purchase Decisions, whereas no significant mediation was observed for Functional Characteristics or Symbolic Characteristics.

**Discussion:**

This research elucidates the “visual priority” tendency among Generation Z consumers. These findings contribute to environmental psychology by clarifying the interplay between sustainability-related perceptions, emotional responses, and pro-environmental consumption.

## 1 Introduction

Amid escalating global climate change and intensifying resource depletion, sustainable development has emerged as a pivotal challenge for human society in the 21st century. In alignment with the United Nations’ 2030 Agenda for Sustainable Development, Responsible Consumption and Production (SDG 12) is prioritized as a key objective, underscoring the imperative to fundamentally transform conventional production and consumption patterns (Lee et al., 2016). Against this backdrop, the manufacturing industry is a major contributor to resource consumption and environmental emissions, which leads to mounting pressure to undergo green transformation.

The textile and apparel industry is widely acknowledged as a highly resource-intensive and polluting sector. The researchers acknowledge that the environmental impact of the textile and apparel industry is a subject of ongoing academic debate. According to the estimates provided by Chen in 2021, this sector accounts for approximately 10 percent of Global Carbon Emissions (Chen et al., 2021). However, that this figure, derived from a 2021 methodological framework based on Global Value Chain (GVC) analysis, varies across different studies. While some reports emphasize the carbon footprint of raw material production, other studies focus more on the energy consumption during manufacturing processes. Therefore, the authors present this 10 percent estimation as a significant but contextualized indicator of the environmental impacts associated with the industry as of the early 2020s. Nonetheless, its environmental footprints, especially the consumption of freshwater resources, remain formidable. Within this sector, denim clothing, which is a cornerstone of global fashion, presents particularly acute environmental challenges. For instance, a United Nations Environment Programme report states that conventional production of a single pair of jeans requires up to 3,000 liters of water (Notten and Nairobi, 2020). Furthermore, research by Abbate indicates that carbon emissions from denim manufacturing account for approximately 8–10% of the total emissions across the global fashion industry (Abbate et al., 2024). Empirical studies have also confirmed that wastewater from denim washing processes frequently exhibits Chemical Oxygen Demand (COD) concentrations between 2,000 and 5,000 mg/L, which are levels that substantially surpass regulatory standards (Polisetty and Pahari, 2025). Against the backdrop of global green and low-carbon development policies, the sustainable transformation of the denim industry has become a matter of increasing urgency.

## 2 Literature review

In recent years, the upcycling of discarded denim fabric has gained significant traction within the industry because it extends the lifecycle of denim through recycling, upgrading, and circular design. Research by the Ellen MacArthur Foundation indicates that circular economy strategies, including the recycling and upcycling of denim clothing, possess the potential to significantly reduce carbon emissions and resource depletion across the product lifecycle. These strategies align closely with the “Cradle to Cradle” concept that is central to sustainable apparel design theory (Ellen MacArthur Foundation, 2017; McDonough and Braungart, 2010). Simultaneously, Generation Z consumers represent a primary market segment and exhibit pronounced “sustainability preference” tendencies in their purchasing behavior. According to Liu’s (2022) findings, 68% of Generation Z consumers express a willingness to pay a 10–15% price premium for garments that feature eco-friendly attributes. Beyond willingness to pay, research has explored the underlying drivers for this cohort. Djafarova and Bowes (2021) highlighted that Gen Z’s purchase intentions are strongly influenced by social media and peer validation, making symbolic and status-driven aspects of sustainability particularly salient. Groening et al. (2018) noted that for Gen Z, environmental concerns are often intertwined with personal identity construction, leading to a “values-to-behavior” gap that is distinct from older generations. Rūtelionė and Bhutto (2024) demonstrated through a serial mediation model that psychological benefits, such as self-esteem and a sense of meaning, mediate the relationship between green apparel attributes and Gen Z’s behavioral intentions. Furthermore, Huh and Kim (2024) examined green signaling effects and found that Gen Z consumers use sustainable fashion as a status symbol to communicate environmental values to their peers. These findings collectively suggest that Gen Z’s engagement with sustainable fashion is not merely a function of environmental awareness but is deeply embedded in their socio-psychological need for self-expression and social belonging. The integration of sustainable concepts into denim clothing, along with efforts to meet the diverse demands of Generation Z consumers for fashion, individuality, and environmental responsibility, has become a significant focus for both academia and the industry.

Current research on sustainable denim clothing primarily diverges into two directions. The technological innovation domain focuses on developing eco-friendly materials and production processes, such as breakthroughs in organic cotton cultivation, water-free dyeing technologies, and bio-enzyme treatment (Hultberg, 2025; Acar et al., 2024; Holmberg et al., 1999). In contrast, consumer behavior studies investigate the extent to which environmental awareness associates Purchase Decisions (Huh and Kim, 2024; Kang et al., 2013). Although these studies have established important theoretical foundations, certain limitations persist. In particular, the psychological mechanisms underlying these relationships require further exploration. From a research perspective, scholars have placed excessive emphasis on technical and economic aspects, which has led to the neglect of intrinsic connections between clothing design elements and consumer emotional experiences. Regarding research subjects, existing literature lacks in-depth analysis specifically targeting Generation Z consumers. This is particularly true concerning their unique emotional response mechanisms and symbolic consumption characteristics. In terms of theoretical construction, previous studies have not adequately explained the mediating roles of Sustainability Values and Emotional Values within the consumption decision-making process.

In the broader context of sustainable consumption research, several theoretical frameworks have been established to address these gaps. The Theory of Planned Behavior (TPB) (Ajzen, 1991) and the Value-Belief-Norm (VBN) theory (Stern, 2000) are among the most prominent. While TPB focuses on the rational determinants of intention such as attitudes and perceived control and VBN emphasizes the alignment of personal values with environmental norms, these frameworks often struggle to capture how specific and tangible product design attributes directly trigger the internal psychological states of consumers. To address this, the Stimulus-Organism-Response (S-O-R) framework offers a more dynamic perspective. Unlike the linear-rational logic of TPB, the S-O-R framework allows for a granular analysis of how external design stimuli categorized here as Appearance Characteristics, Functional Characteristics, and Symbolic Characteristics elicit internal cognitive and affective mediators, namely Sustainability Values and Emotional Values, which subsequently drive Purchase Decisions. By adopting the S-O-R framework, this study provides a more precise reflection of the perceptual-driven consumption patterns characteristic of Generation Z.

Kansei Engineering is a methodology that links product attributes with user emotional experiences (Nagamachi, 1995; Nagamachi, 2016). This approach demonstrates unique value in the field of sustainable design. When utilized as a verification tool (Nagamachi, 1995), Structural Equation Modeling (SEM) can be integrated with Bootstrap mediation analysis (Nagamachi, 2016) to reveal how design elements are associated with Purchase Decisions through environmental and emotional pathways. These methodologies provide a solid foundation for constructing an innovative “Kansei Engineering-SEM-Bootstrap” research framework. This system effectively addresses the demand for analyzing the emotional response mechanisms of Generation Z consumers.

Initially, this study utilizes Kansei Engineering theory to systematically extract the Kansei image vocabulary perceived by Generation Z consumers. These terms are then transformed into quantifiable observed variables, which include Appearance, Functional, and Symbolic Characteristics. Secondly, Structural Equation Modeling (SEM) is employed to verify the complex path relationships between latent variables and Purchase Decisions. The researchers conduct exploratory factor analysis and confirmatory factor analysis to ensure the validity of the measurement model. Furthermore, the study applies the Bootstrap mediating effect test to analyze the dual mediation mechanism involving Sustainability Values and Emotional Values. Finally, this paper proposes an innovative design strategy for denim clothing that integrates sustainability principles with emotional design, drawing upon the findings from the empirical study. This research provides theoretical foundations and practical guidance for the green transformation of denim clothing. Additionally, it offers new insights into balancing eco-friendly attributes with market demands. This study contributes to the field of Environmental Psychology by examining how cognitive and affective processes are associated with pro-environmental consumption behavior.

## 3 Methods

### 3.1 Theoretical integration

In order to rigorously contextualize the psychological mechanisms that related to sustainable consumption, this study integrates the Stimulus-Organism-Response (S-O-R) framework with the Value-Belief-Norm (VBN) theory. Within this model, the physical and symbolic attributes of apparel, including Appearance, Functionality, and Symbolism, act as environmental stimuli (Stimulus). These stimuli are associated with the internal cognitive and affective evaluations of consumers. Specifically, these evaluations encompass perceived Sustainability Values and Emotional Values (Organism), which are associated with behavioral intentions, specifically the Purchase Decisions (Response). The combination of these environmental psychology theories and Kansei Engineering allows this study to effectively address the literature gap regarding the internal emotional mechanisms of Generation Z consumers.

Kansei Engineering, Structural Equation Modeling (SEM), and Bootstrap mediation analysis constitute the three core methodological foundations of this study. Kansei Engineering utilizes the Semantic Differential Method to translate the subjective emotional needs of consumers into quantifiable design elements (Ajzen, 1991). In addition, SEM is employed to verify complex path relationships among variables. The hypothesis-driven nature of this method offers a rigorous analytical framework for the research (Ahmed et al., 2024). Furthermore, Bootstrap mediation analysis utilizes resampling techniques to assess the strength of mediating variables with high precision (Streukens and Leroi-Werelds, 2016). These three methods complement each other effectively. Together, they form a comprehensive methodological framework for this study.

#### 3.1.1 Theoretical foundation

Structural Equation Modeling (SEM) is a hypothesis-driven multivariate statistical method (Ahmed et al., 2024). The analytical process of this method consistently revolves around the formulation and verification of theoretical hypotheses. Therefore, researchers must propose clear path hypotheses based on theoretical foundations during the model construction phase. This study employs SEM in conjunction with Bootstrap mediation analysis. The goal is to investigate the mechanisms through which product appearance, functionality, and Symbolic Characteristics operate based on product design theory. Furthermore, this study draws upon consumer behavior theory to examine the relationships among Sustainability Values, Emotional Values, and Purchase Decisions. Specifically, the authors propose 11 direct path hypotheses and six mediation hypotheses.

##### 3.1.1.1 Direct effect hypotheses

This study synthesizes recent research findings within the sustainable consumption domain to develop a theoretical model explaining the Purchase Decisions of consumers regarding sustainable denim clothing. The model incorporates five key variables. These include Appearance Characteristics, Functional Characteristics, Symbolic Characteristics, Sustainability Values, and Emotional Values. On this basis, the authors propose the following hypotheses regarding direct effects.

###### 3.1.1.1.1 Appearance characteristics

Product appearance represents the first impression formed by consumers and plays a crucial role in the purchase decision-making process. According to Bloch’s product form attractiveness theory, the aesthetic features of a product directly are associated with consumer attitudes and behavioral intentions (Bloch, 1995). In the context of apparel consumption, Reimann et al. (2010) utilized eye-tracking experiments to confirm that consumer evaluations of clothing are formed within the initial 7 seconds, wherein visual attractiveness serves as a critical determining factor. Regarding sustainable fashion products, Joung and Park-Poaps (Joung et al., 2013) identified a significant “visual priority” tendency among Generation Z consumers, who prefer garments that maintain design appeal while preserving eco-friendly attributes. The concept of “aesthetic sustainability” proposed by Niinimäki (2013) explains the relationship between product appearance and Sustainability Values, suggesting that superior design can enhance consumer perceptions of product sustainability. Drawing upon Norman’s (Norman and Ortony, 2003) three-level theory of emotional design, Appearance Characteristics not only shape consumer responses at the visceral level but also enhance emotional experiences through the reflective level. Consequently, the authors propose the following hypotheses:

*H11:* Appearance Characteristics are positively associated with Purchase Decisions.

*H21:* Appearance Characteristics are positively associated with Sustainability Values.

*H31:* Appearance Characteristics are positively associated with Emotional Values.

###### 3.1.1.1.2 Functional characteristics

Product Functional Characteristics represent a core dimension utilized by consumers during rational evaluations. Utility Maximization Theory (Lancaster, 1966) posits that consumers evaluate the utilitarian value of products based on their objective attributes. In the context of sustainable clothing, Kang et al. (2013) argue that functional attributes including durability, comfort, and applicability significantly are associated with the assessment of Sustainability Values among consumers. Research by Allwood et al. (2008) utilizes life cycle assessment to confirm that clothing products with extended service lives provide greater environmental benefits. However, when evaluating product functionality, Generation Z consumers frequently prioritize the emotional experience linked to functional attributes over practical value. This shift increases the complexity of the mechanism through which Functional Characteristics impact Emotional Values. Based on these premises, the authors propose the following hypotheses:

*H12:* Functional Characteristics are positively associated with Purchase Decisions.

*H22:* Functional Characteristics are positively associated with Sustainability Values.

*H32:* Functional Characteristics are positively associated with Emotional Values.

###### 3.1.1.1.3 Symbolic characteristics

Symbolic Characteristics occupy a significant position within Consumer Behavior research. According to Symbolic Consumption Theory (Ekinci et al., 2013), modern consumption represents a form of symbolic behavior. Through this process, consumers construct and express their Self-identity via product choices. In the field of sustainable consumption, Griskevicius et al. (2010) found that products with environmental symbolic meaning can fulfill the status display needs of consumers, a phenomenon referred to as “Conspicuous Conservation.” Furthermore, White et al. (2019) indicated that the relationship between brand symbolic value and green product purchase intentions demonstrates significant generational differences. In this context, Generation Z consumers demonstrate the highest sensitivity to brand values. However, current research presents conflicting findings regarding the relationship between Symbolic Characteristics and Emotional Values. For instance, some scholars argue that brand symbolic meaning is positively associated with Emotional Connection (Belk, 1988). In contrast, other studies report that this effect is insignificant among younger consumer groups. Based on Social Identity Theory (Tajfel and Turner, 1979), this paper hypothesizes that brand Symbolic Characteristics are positively associated with consumer Emotional Values. Consequently, the authors propose the following hypotheses.

*H13:* Symbolic Characteristics are positively associated with Purchase Decisions.

*H23:* Symbolic Characteristics are positively associated with Sustainability Values.

*H33:* Symbolic Characteristics are positively associated with Emotional Values.

###### 3.1.1.1.4 Sustainability values

Sustainability Values is acknowledged as a core driving force within the domain of Sustainable Consumption research. According to Norm Activation Theory (Schwartz, 1977), individuals develop a sense of moral obligation when they perceive environmental issues to be aligned with their personal values. This internal state in turn is associated with pro-environmental behavior. Consequently, the authors propose the following hypothesis.

*H4:* Sustainability Values has a positive effect on Purchase Decisions.

###### 3.1.1.1.5 Emotional values

The role of Emotional Values within the Consumption Decision-making Process is gaining significant attention. Affect Primacy Theory (Zajonc, 1980) posits that emotional responses can occur within 100 milliseconds following the presentation of a stimulus. These responses often precede and function independently of the Cognitive Appraisal Process. Positive emotional experiences exert a positive impact on Purchase Decisions. Consequently, the authors propose the following hypothesis.

*H5:* Emotional Values has a positive effect on Purchase Decisions.

##### 3.1.1.2 Mediating effect hypothesis

The researchers conducted in-depth interviews with 37 Generation Z Consumers to identify the Mediating Variables for the conceptual model. The authors utilized snowball sampling methods and purposive sampling methods to recruit participants born between 1995 and 2009 who demonstrated high levels of Environmental Awareness. The researchers performed these Interviews either in person or via video conferencing platforms, with each session lasting between 45 and 60 min. The authors designed the Interview Protocols around three question themes, including Consumption Motivations, Perceived Values of sustainable denim, and Emotional Connections with Green Products.

To process the qualitative data, the researchers employed thematic analysis methods and grounded theory approaches. The authors transcribed the audio recordings and performed three levels of Coding Procedures, namely open coding, axial coding, and selective coding. The results revealed that Sustainability Values and Emotional Values emerged as the most frequently mentioned psychological constructs that link Product Attributes to Purchase Decisions. Specifically, the researchers identified Sustainability Values as a Mediating Construct because the participants consistently emphasized the importance of environmental responsibilities and resource consumptions. Furthermore, the authors designated Emotional Values as a second Mediating Construct based on the recurring Participant Narratives regarding the psychological satisfactions and self-Identities derived from wearing sustainable apparel. This rigorous qualitative approach provides a solid Theoretical Foundation for the Mediating Effects hypothesized in the Structural Equation Model. Based on the theoretical framework and the preliminary qualitative findings, the authors propose the following mediation hypotheses.

*Ha:* Sustainability Values mediate the relationship between Appearance Characteristics and Purchase Decisions.

*Hb:* Sustainability Values mediate the relationship between Functional Characteristics and Purchase Decisions.

*Hc:* Sustainability Values mediate the relationship between Symbolic Characteristics and Purchase Decisions.

*Hd:* Emotional Values mediate the relationship between Appearance Characteristics and Purchase Decisions.

*He:* Emotional Values mediate the relationship between Functional Characteristics and Purchase Decisions.

*Hf:* Emotional Values mediate the relationship between Symbolic Characteristics and Purchase Decisions.

#### 3.1.2 Theoretical model construction

This study utilizes Kansei Engineering Theories to integrate both qualitative attributes and quantitative attributes within apparel design research. Consequently, the researchers selected the semantic differential methods within the framework of Kansei Engineering as the primary research methodologies. To capture the genuine perceptions of Generation Z Consumers, the authors recruited a participant panel consisting of 50 university students through purposive sampling methods via university online forums. The demographic characteristics of the participants included a balanced distribution consisting of 26 females and 24 males, all of whom were born between 1995 and 2009.

Based on a literature review (Lixu et al., 2021; Banytė et al., 2023; Acar et al., 2024; Nicolau et al., 2025; Joshi and Rahman, 2017) and initial user surveys, the researchers identified 30 sets of candidate Kansei Word Pairs. The authors administered a preliminary questionnaire to the 50 participants to ensure the scientific validity of the selection. During this process, the participants evaluated the relevance of each pair using a 7-point Likert Scale. The researchers applied a selection criterion that excluded Items with mean relevance scores lower than 4.0, which effectively reduced the initial pool from 30 to 20 Kansei Word Pairs. The researchers intentionally selected a 7-point Likert Scale rather than a 5-point Likert Scale to increase the sensitivity of the measurement instrument. Since the survey target consisted of general consumer groups, the authors aimed to capture more nuanced psychological gradients and subtle differences in consumer attitudes. The researchers assert that a 7-point Likert Scale provides a broader range of options, which reduces the tendency of respondents to select neutral midpoint answers and improves the overall Reliability of the Statistical Analyses.

Subsequently, the researchers invited six expert judges, including three senior faculty members and three industry professionals, to evaluate these 20 Items based on semantic distinctness and their alignment with garment design principles. The authors then selected the final 12 Kansei Word Pairs through a consensus-building process, requiring a minimum of 80 percent agreement among the judges to ensure professional validity. These 12 Items were then allocated to the three Independent Variable Dimensions, namely Appearance Characteristics, Functional Characteristics, and Symbolic Characteristics. The researchers assigned four specific Items to each dimension based on their primary semantic focus, ensuring that each construct was measured by a distinct and theoretically relevant set of descriptors to facilitate the subsequent Statistical Analyses. These results, which form the empirical basis for understanding Purchase Decisions, are presented in Table 1.

**Table 1:** Kansei word pairs.

Number	Kansei word pairs	Number	Kansei word pairs
1	Loose–Fitted	11	Neat–Chaotic
2	Flexible–Rigid	12	Eye-catching–Understated
3	Avant-garde–Conservative	13	Fashionable–Plain
4	Diverse–Singular	14	Rich–Monotonous
5	Practical–Decorative	15	Harmonious–Discordant
6	Innovative–Traditional	16	Versatile–Unique
7	Exquisite–Rough	17	Compact–Independent
8	Personalized–Mass–Oriente	18	Comfortable–Uncomfortable
9	Unrestrained–Constrained	19	Eco-friendly–Harmful
10	Multi-functional–Limited	20	Textured–Flat

The researchers invited six experts with over 10 years of experience in fashion design to organize, classify, and associate the collected Kansei Words with clothing elements. Eventually, the expert panel formed 12 pairs of Kansei Words including “Fashionable and Plain,” “Harmonious and Discordant,” “Loose and Fitted,” and “Eye-catching and Understated.” The experts then matched these pairs with Appearance Characteristics, Functional Characteristics, and Symbolic Characteristics. These mappings are presented in Table 2. The participants conducted the classification through consensus discussion based on semantic similarity and design relevance.

**Table 2:** Independent variable and emotional image word pair.

Independent variable	Emotional image word pair
Appearance characteristics Functional characteristics Symbolic characteristics	“Fashionable-Plain,” “Harmonious-Discordant” “Loose-Fitted,” “Eye-catching-Understated” “Multi-functional-Limited,” “Versatile-Unique” “Practical-Decorative,” “Comfortable-Uncomfortable” “Unrestrained-Constrained,” “Personalized-Mass-oriented” “Compact-Independent,” “Diverse-Singular”

The researchers converted the emotional image word pairs corresponding to the Independent Variables in Table 2 into academic terminology. This process resulted in 12 s-order dimensions of Observed Variables, including “Stylish Design,” “Harmonious Color,” “Loose Silhouette,” and “Eye-catching Pattern” (Nicolau et al., 2025; Joshi and Rahman, 2017; Budi et al., 2024). The authors then matched these second-order dimensions with the three first-order dimensions of Independent Variables, namely Appearance Characteristics, Functional Characteristics, and Symbolic Characteristics. These mappings are presented in Table 3.

**Table 3:** Observation indicator system.

Latent variables (first-order dimension)	Item number	Observed variables (second-order dimension)	References
Appearance characteristics	AC1 AC2 AC3 AC4	Stylish design Harmonious color Loose silhouette Eye-catching pattern	Budi et al. (2024)
Functional characteristic	FC1 FC2 FC3 FC4	Removable design Wide application Utility pockets Casual and comfortable	Park and Yim (2013)
Symbolic characteristic	SC1 SC2 SC3 SC4	Cultural symbolism Individual expression Social connectivity Multicultural harmony	Kwon and Lee (2015)
Sustainability values	SV1 SV2 SV3	Long-lasting and durable Recycling of second-hand clothing re-manufacturing of jeans	Kwan (2012) and Kim et al. (2021)
Emotional values	EV1 EV2 EV3	Social value Hedonic value Psychological value	Rūtelionė and Bhutto (2024)
Purchase decisions	PD1 PD2 PD3 PD4	Design satisfaction Purchase intention Wear intention Recommendation intention	Benhissi and Hamouda (2025)

**Figure 1:**
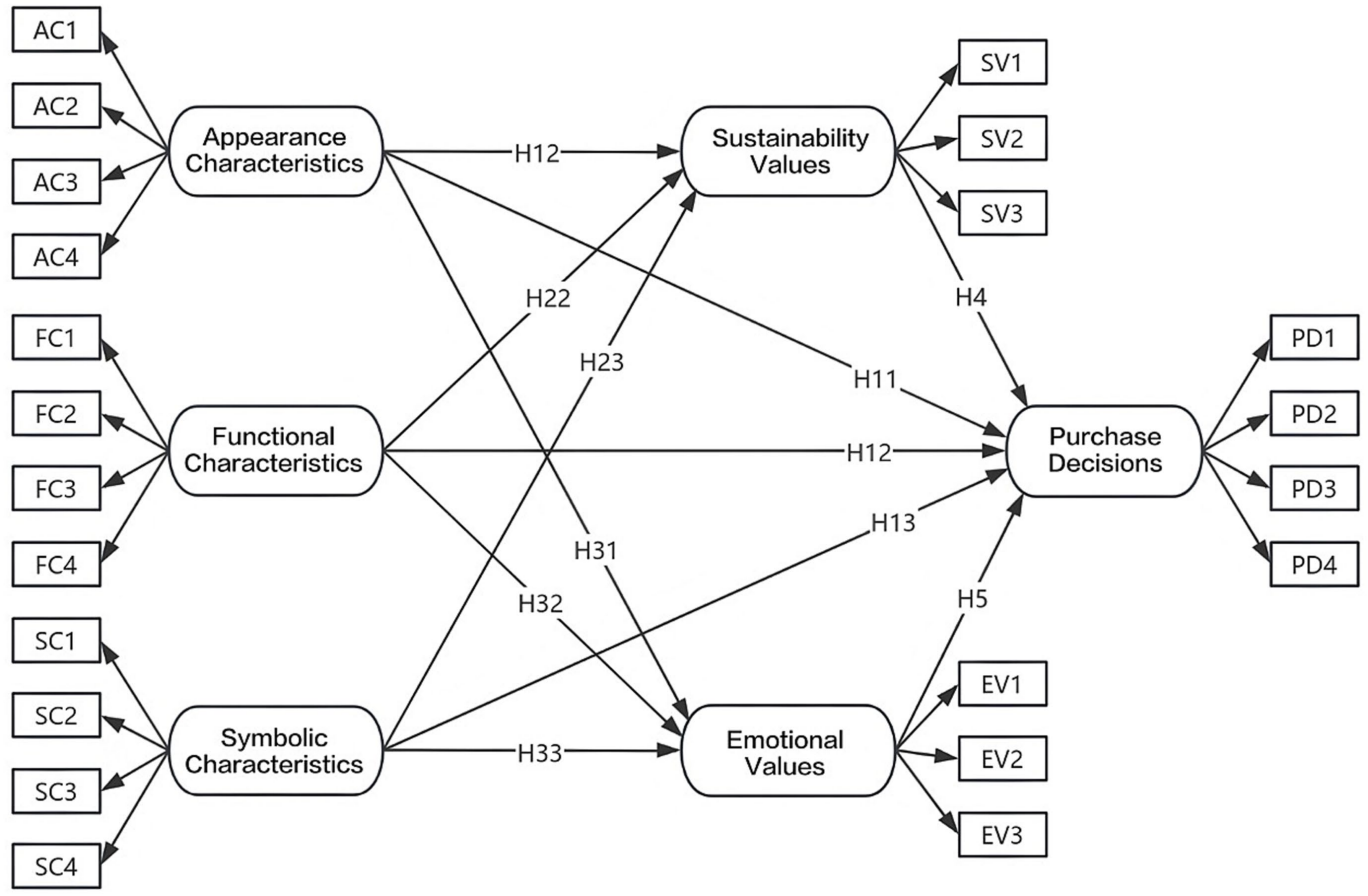
Theoretical model of influencing factors on sustainable denim purchase decisions.

Based on a systematic review of existing literature (Guo et al., 2024; Rūtelionė and Bhutto, 2024; Kwan, 2012; Kim et al., 2021; Jiang et al., 2024; Peterson et al., 2021; Benhissi and Hamouda, 2025), the researchers further identified the Mediating Variables and the Observed Variables of the Dependent Variables. The authors constructed a model that not only aligns with the disciplinary characteristics of fashion design but also ensures the scientific rigor of the research process and the reliability of the research results. The researchers presented the Theoretical Model in Figure 1.

### 3.2 Scale design and data collection

#### 3.2.1 Scale design

The researchers employed a three-round Delphi Method to ensure the Content Validity of the initial Survey Items. The authors invited a panel of six expert judges, including three senior faculty members specializing in Sustainable Fashion and three industry professionals with over 5 years of experience in denim design.

In the first round, the researchers distributed an open-ended questionnaire to the experts to evaluate the initial 20 Kansei Word Pairs derived from the previous screening stage. The results revealed initial disagreements regarding the semantic boundaries of Symbolic Characteristics and Sustainability Values, with an initial consensus rate of only 65 percent. Specifically, several experts suggested refining the descriptions of fabric textures to avoid conceptual overlap with Appearance Characteristics.

To reach a statistical consensus in the second round, the researchers applied a strict consensus threshold requiring a minimum of 75 percent agreement among the judges, alongside a Content Validity Index (CVI) greater than 0.80 and a coefficient of variation less than 0.25. The authors removed eight Items during this stage because these Items were deemed semantically ambiguous or shared significant conceptual overlap with Emotional Values. For instance, the researchers deleted four specific descriptors that failed to distinguish between sensory aesthetics and psychological pleasure to ensure the discriminant validity of the measurement instrument. Furthermore, the experts recommended refining the linguistic nuances of the remaining items to better capture the specific Sustainability Values relevant to the Generation Z market.

In the third Round, the researchers presented the revised list of 12 final Kansei Word Pairs to the Panel. The authors observed that the mean importance scores for all remaining Variables exceeded 6.0 on a 7-point Likert Scale, and the final consensus rate exceeded 85 percent as the coefficient of variation dropped below 0.15. These figures indicated a high degree of expert consensus. Consequently, the researchers finalized the measurement instrument, ensuring that the four retained Items for each dimension accurately reflected the Appearance Characteristics, Functional Characteristics, and Symbolic Characteristics of sustainable denim clothing. These systematic refinements provided a robust empirical foundation for the subsequent analysis of Purchase Decisions. The researchers presented the finalized Measurement Indicator System in Table 4.

**Table 4:** Measurement items.

Latent variables	Item of observed variables
Appearance characteristics	AC1: I believe that fashionable styles significantly associated my purchase of sustainable denim clothing. AC2: I find sustainable denim clothing with harmonious colors more appealing. AC3: I prefer to purchase sustainable denim clothing featuring a loose silhouette. AC4: I’m more likely to buy sustainable denim clothing if it has eye-catching patterns.
Functional characteristics	FC1: I think removable design is an added advantage of sustainable denim clothing. FC2: I believe versatility is the primary factor in my purchase of sustainable denim clothing. FC3: I prefer to choose sustainable denim clothing with utility pockets (multi-pocket functionality). FC4: I consider casual comfort a key factor influencing my purchase of sustainable denim clothing.
Symbolic characteristics	SC1: I see sustainable denim clothing as an eco-friendly, liberating cultural symbol. SC2: I feel that wearing sustainable denim clothing strongly reflects individuality. SC3: Sustainable denim clothing helps me connect with environmental advocates. SC4: I like buying clothes that blend sustainability with denim culture.
Sustainability values	SV1: I prefer to buy clothes that are durable. SV2: I should buy sustainable denim clothing to support national environmental policies. SV3: I desire to purchase eco-friendly denim clothing that is stylish and has aesthetic design value.
Emotional values	EV1: Wearing sustainable denim clothing is associated with my sense of social responsibility and belonging. EV2: Wearing sustainable denim clothing brings me both aesthetic pleasure and emotional satisfaction. EV3: I believe wearing sustainable denim clothing shows environmental concern, easing my guilt.
Purchase decisions	PD1: My satisfaction with the design of sustainable denim clothing will associated my Purchase Decisions. PD2: I intend to purchase sustainable denim clothing to contribute to national environmental efforts. PD3: I would wear sustainable denim clothing in my daily life. PD4: I am willing to recommend sustainable denim clothing to friends or family members.

#### 3.2.2 Data collection

This study employed a multi-source convenience sampling strategy. The researchers conducted data collection from January 2025 to April 2025. The authors implemented a Digital Opt-in Consent Procedure to adhere to strict ethical standards. The researchers suggest that the findings should be interpreted with caution regarding Generalizability because of the non-probability nature of this approach. All participants read an informational page detailing the purpose of the study before clicking to agree and proceeding to the survey. This document assured complete anonymityas the researchers recorded no IP addresses or identifiable data.

Regarding the definition of Generation Z, the researchers acknowledge that academic institutions hold divergent views on the exact age boundaries. While some researchers define Generation Z as individuals born after 1997, the authorsadopted a classification starting from 1995. The researchers based this decision on the study conducted by Priporas, Stylos, and Fotiadis in 2017, who define Generation Z as individuals born after 1995 and emphasize their unique status as the first generation of true digital natives (Priporas et al., 2017). Although the sample includes individuals born between 1995 and 1999 who are strictly classified as Older Millennials, these individuals share highly similar socio-cultural and digital consumption habits with Generation Z in the Chinese context. Consequently, the authors retained these respondents in the analysis.

To ensure compliance with international research ethics standards and protect the rights of participants, rigorous ethical protocols were implemented during the data collection process. Prior to accessing the formal survey items, all participants were presented with a mandatory Digital Informed Consent page. This page provided comprehensive information regarding the study, including: (1) the academic purpose of the research, which focuses on how product attributes influence the Purchase Decisions of Generation Z; (2) the voluntary nature of participation and the explicit right to withdraw from the survey at any point without any negative consequences; and (3) the estimated time required for completion.

Formal consent was obtained through a “Digital Opt-in” procedure, where participants were required to click a confirmation button (“I have read the above information and voluntarily agree to participate”) to proceed to the questionnaire. This electronic affirmation served as the informed consent, replacing traditional written signatures to maintain the efficiency and reach of the online survey. Furthermore, strict measures were taken to ensure the anonymity of all responses. No personally identifiable information (PII), such as names, contact details, or IP addresses, was collected or stored. The survey platform was configured to process data in an encrypted and aggregated format, ensuring that individual perspectives on Appearance Characteristics, Emotional Values, and Sustainability Values could not be traced back to any specific respondent. All data were used solely for academic analysis, thereby safeguarding the privacy and confidentiality of the Generation Z consumers involved in this study.

Besides, the authors addressed the ethical considerations regarding the younger participants. For the respondentsborn in 2009 who were approximately 15 to 16 years old during the data collection, the researchers strictly adhered to ethical guidelines for Informed Consent. The authors obtained explicit Informed Consent from both the adolescent participants and their legal guardians before the commencement of the survey. These procedures ensured that all participants understood the voluntary nature of the study and the confidentiality of their personal information.

The survey results revealed a nearly equal gender distribution. Male respondents numbered 220, accounting for 50.93 percent. Female respondents numbered 212, representing 49.07 percent. The age distribution clearly reflected generational characteristics. The largest cohort consisted of 202 individuals born between 2000 and 2004, which represented 46.76 percent of the sample. This group was followed by 124 individuals born between 2005 and 2009, accounting for 28.70 percent. The smallest proportion originated from those born between 1995 and 1999, representing 24.54 percent with 106 individuals.

Regarding educational backgrounds, respondents with bachelor’s or associate degrees formed the largest group, comprising 198 individuals or 45.83%. This group was followed by 132 individuals with high school or lower education levels, representing 30.56%. respondents holding master’s degrees or higher accounted for a smaller share of 102 individuals at 23.61%. The monthly consumption levels show a stepwise distribution. The group with a monthly consumption of RMB 3000 or less accounts for more than half of the sample, totaling 230 individuals. The group consuming between RMB 3001 and 7,000 accounts for 29.17% with 126 individuals, while those spending between RMB 7001 and 15,000 make up 13.66% with 59 individuals. The smallest proportion is represented by the high-consumption group exceeding RMB 15,001, which includes 17 individuals.

The occupational distribution reveals that students constitute half of the sample. This group includes 216 individuals. Employees in private enterprises follow this group with 115 individuals, representing 26.62%. Smaller proportions are represented by 59 staff members working in government agencies or public institutions and 42 self-employed individuals. The authors presented the detailed data in Table 5. Overall, the sample demonstrates good diversity and balance in demographic characteristics. The broad distribution across various dimensions indicates strong representativeness.

**Table 5:** Descriptive statistical analysis of demographic variables.

Variable	Option	Number	Percentage (%)
Gender	Male	220	50.926
	Female	212	49.074
Age	1995–1999	106	24.537
	2000–2004	202	46.759
	2005–2009	124	28.704
Education level	High School or Lower	132	30.556
	University Bachelor’s or Associate Degree	198	45.833
	Master’s Degree or Higher	102	23.611
Monthly consumption level	RMB 3,000 or Below	230	53.241
	RMB 3,001–7,000	126	29.167
	RMB 7,001–15,000	59	13.657
	RMB 15,001 or Above	17	3.935
Occupation	Student	216	50.000
	Government and Public Institution Employee	59	13.657
	Private Sector Employee	115	26.620
	Freelancer	42	9.722

### 3.3 Common method bias testing

The researchers addressed the potential for Common Method Bias resulting from the use of a single self-report instrument. Following the procedural recommendations of Podsakoff et al. (2003), the researchers implemented several Procedural Controls, such as ensuring respondent anonymity and clarifying that there were no right or wrong answers to reduce Evaluation Apprehension.

Furthermore, the authors conducted Harman’s Single-Factor Test to statistically assess the Common Method Variance. The results of the Exploratory Factor Analysis showed that the first principal component accounted for only 32.4 percent of the total variance. Since this value is well below the 50 percent threshold, the researchers confirmed that no single factor emerged as a dominant source of variance. Therefore, the authors concluded that Common Method Bias does not pose a significant threat to the validity of the Hypothesis Testing in this study.

### 3.4 Model testing and empirical analysis

#### 3.4.1 Common method bias and normality check

Given the cross-sectional and self-reported nature of the data, the researchers conducted Harman’s Single-factor Test in SPSS to check for Common Method Variance. The Unrotated Principal Component Analysis revealed that the first factor accounted for 36.4% of the total variance. This value remains well below the conservative 40% threshold. These results indicate that Common Method Variance does not significantly bias the findings. Consequently, Common Method Bias is unlikely to pose a serious threat to the validity of the study.

To justify the use of Maximum Likelihood (ML) estimation in AMOS, the researchers examined the univariate normality of all observed indicators. The skewness values for the 22 items ranged from −2.569 to −0.261, and the kurtosis values ranged from −0.237 to 9.104 in Table 6. According to the strict thresholds proposed by Kline (2015), |skewness| < 2, |kurtosis| < 7, a few items (e.g., AC1 with skewness = −2.569, kurtosis = 8.617; SV3 with kurtosis = 9.104) showed moderate deviations from univariate normality. However, more flexible guidelines Kline (2023), Hair et al., 2010 (Hair et al., 2010) suggest that absolute skewness values below 3 and absolute kurtosis values below 10 are acceptable for structural equation modeling. When the sample size (*N* ≥ 200), even if skewness and kurtosis slightly exceed the recommended ranges, the impact on SEM results based on Maximum Likelihood (ML) estimation is minimal and generally not a cause for concern. Therefore, the data (*N* = 432 in this study are considered suitable for ML estimation.

**Table 6:** Descriptive statistics of variables.

Variable	N	Minimum	Maximum	Mean	Standard deviation	Skewness	Kurtosis
AC1	432	1	7	6.090	1.162	−2.569	8.617
AC2	432	1	7	5.991	1.062	−2.259	7.503
AC3	432	1	7	6.035	1.182	−2.395	7.765
AC4	432	1	7	5.914	1.182	−2.181	6.87
FC1	432	1	7	5.938	0.996	−0.994	1.435
FC2	432	1	7	5.944	1.029	−1.363	2.578
FC3	432	1	7	5.861	1.008	−1.099	1.951
FC4	432	1	7	5.456	1.012	−0.697	0.775
SC1	432	1	7	5.523	1.201	−0.261	−0.841
SC2	432	1	7	5.447	1.307	−0.725	−0.237
SC3	432	1	7	5.463	1.279	−0.675	0.002
SC4	432	1	7	5.046	1.257	−0.264	−0.685
SV1	432	1	7	6.169	0.922	−2.256	6.924
SV2	432	1	7	6.093	0.899	−2.153	6.535
SV3	432	1	7	6.063	0.885	−2.498	9.104
EV1	432	1	7	6.176	1.065	−1.429	3.314
EV2	432	1	7	6.208	1.010	−1.608	4.558
EV3	432	1	7	6.215	1.000	−1.355	3.938
PD1	432	1	7	6.278	0.841	−1.785	6.038
PD2	432	1	7	6.192	0.819	−1.488	5.295
PD3	432	1	7	6.262	0.761	−1.75	7.714
PD4	432	1	7	6.215	0.799	−1.861	7.822

To further ensure the robustness of the inferences, the researchers employed the Bootstrap procedure with 5,000 resamples to compute bias-corrected confidence intervals and robust standard errors for all path coefficients and indirect effects. The bootstrap method does not rely on the normality assumption (Nevitt and Hancock, 2001; Pesigan and Cheung, 2020) and provides a stringent test of the stability of the estimates. The bootstrap results confirmed the significance and direction of all hypothesized effects reported in the main analysis, indicating that the mild deviations from normality do not substantively affect the conclusions.

#### 3.4.2 Reliability analysis

The researchers employed SPSS 22.0 statistical software to conduct Reliability Tests on the questionnaire data. The authors utilized the Cronbach’s *α* Coefficient as the core measure of Internal Consistency (Nunnally, 1975). According to Psychometric Standards, an *α* Coefficient between 0.7 and 0.9 indicates good reliability. Furthermore, an *α* Coefficient greater than or equal to 0.9 indicates excellent reliability (Cronbach, 1951). The researchers presented these results in Table 7. The Cronbach’s *α* Coefficients for all Latent Variables remain substantially above the minimum threshold of 0.7.

**Table 7:** Reliability analysis.

Latent variables	Number	Cronbach’s *α*
Appearance characteristics	4	0.921
Functional characteristics	4	0.897
Symbolic characteristics	4	0.944
Sustainability values	3	0.867
Emotional values	3	0.904
Purchase decisions	4	0.908

Further analysis shows that the Corrected Item-total Correlation for every item exceeds the recommended cutoff value of 0.5 (Murtagh and Kurtz, 2016). The results also indicate that the removal of any single item does not increase the *α* Coefficient compared to the original dimension. This pattern indicates a highly stable internal structure for the scale. These findings confirm that the Latent Variables demonstrate excellent Reliability. This performance effectively reflects the model assumptions and establishes a solid basis for subsequent empirical analyses.

#### 3.4.3 Validity analysis

In empirical analysis, the researchers define Validity as the extent to which measurement outcomes accurately predict Latent Variables (Bai et al., 2024). The authors adopted a Combined Validation Strategy employing Exploratory Factor Analysis (Watkins, 2018) and Confirmatory Factor Analysis (Harrington, 2009) to ensure the scientific validity and reliability of the test. In this approach, the researchers primarily utilized Exploratory Factor Analysis to explore the Structural Validity of the model. Meanwhile, the authors utilized Confirmatory Factor Analysis to assess the Goodness-of-fit of the predefined factor structure. These two methods complement each other and the researchers collectively enhanced the rigor of the Validity Test through this integration.

##### 3.4.3.1 Exploratory factor analysis

The Exploratory Factor Analysis begins with a Feasibility Assessment. This assessment must satisfy two primary criteria. First, the Kaiser-Meyer-Olkin Measure should exceed 0.7. Second, the Bartlett’s Test of Sphericity should yield a statistically significant result. This significance is defined by a *p*-value of less than 0.05. The researchers conducted the Kaiser-Meyer-Olkin Measure and the Bartlett’s Test of Sphericity using SPSS 22.0 to evaluate the suitability of the rating scale for Factor Analysis.

The researchers presented the results in Table 8. The Kaiser-Meyer-Olkin Value reached 0.898, which surpasses the recommended threshold of 0.8. This result indicates strong inter-correlations among Variables and confirms the appropriateness of the dataset for Factor Analysis (Kaiser, 1974). The Bartlett’s Test of Sphericity yielded an approximate Chi-square Statistic of 7534.683. The degrees of freedom were 231, and the *p*-value was less than 0.001. This statistical significance further supports the presence of sufficient Common Variance among Variables and fulfills the fundamental assumptions of Factor Analysis (Field and Gillett, 2010). For further analysis, the authors employed Principal Component Analysis to extract factors with Eigenvalues greater than 1. Meanwhile, the researchers applied Varimax Orthogonal Rotation to facilitate interpretation during Factor Rotation. Twenty-two Items had Factor Loadings greater than 0.5 across six Factors. The cumulative explained variance exceeded 60%. Consequently, the authors consider the validity of the external factors scale to be reliable.

**Table 8:** KMO and Bartlett’s test of sphericity.

Indicator category		Number
KMO measure of sampling adequacy		0.898
Bartlett’s test of sphericity	Approximate chi-square	7,534.683
	Degrees of freedom	231
	Significance	0.000

The researchers conducted Principal Component Analysis to examine the dimensional structure of the measurement instrument. Based on the Kaiser Criterion requiring Initial Eigenvalues to exceed 1, the authors extracted six Principal Components. The researchers present the statistical details of this extraction in Table 9. The cumulative variance explained reached 81.176 percent, which significantly exceeds the common academic threshold of 60 percent. This high percentage confirms that the model effectively captures the information contained in the Original Variables. To facilitate the interpretation of these factors, the authors applied Varimax Orthogonal Rotation during Factor Rotation. The researchers identified that twenty-two Items possessed Factor Loadings greater than 0.5 across the six Factors. After rotation, the authors observed individual Variance Contribution Rates of 15.789 percent, 14.814 percent, 14.216 percent, 14.200 percent, 11.217 percent, and 10.939 percent, respectively. These figures suggest a relatively robust Measurement Structure and demonstrate the excellent Multidimensionality and reliability of the External Factors Scale.

**Table 9:** Total variance explained for the principal components.

Component	Initial eigenvalues (total)	Variance contribution rates (percent)	Cumulative variance (percent)
1	8.400	15.789	15.789
2	3.139	14.814	30.603
3	1.986	14.216	44.819
4	1.803	14.200	59.019
5	1.380	11.217	70.236
6	1.151	10.939	81.176

##### 3.4.3.2 Confirmatory factor analysis

The authors utilized Confirmatory Factor Analysis as a statistical method to assess whether the Measurement Modelling with theoretical assumptions. Confirmatory Factor Analysis aims to verify whether the relationship between the prespecified Factor Structures and the Observed Variables meets theoretical expectations. The following section evaluates the Measurement Models through two primary aspects, namely Structural Validity and Convergent Validity.

###### 3.4.3.2.1 Structural validity

**Figure 2:**
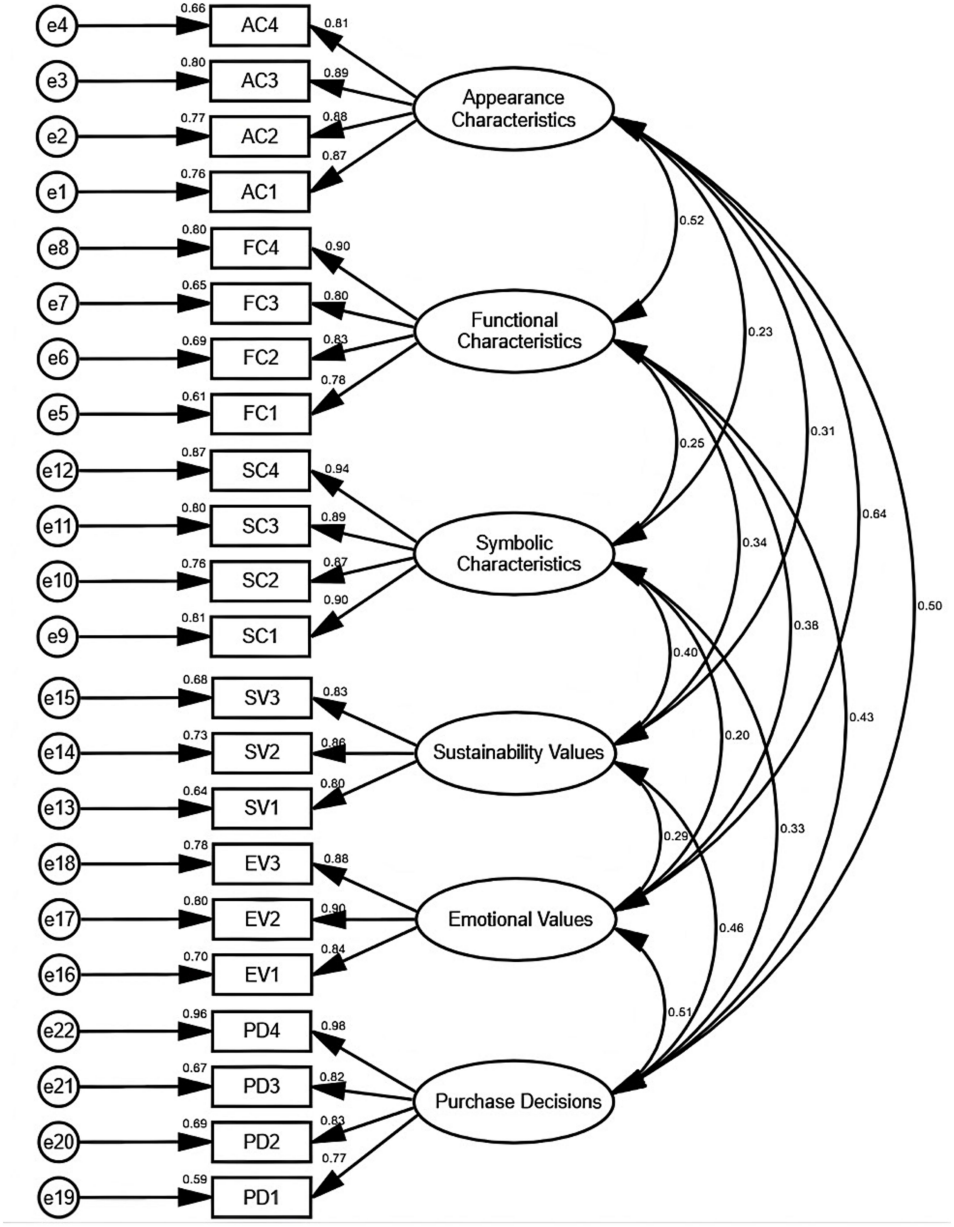
Confirmatory factor analysis path coefficients.

Structural Validity reflects the consistency between the Measurement Models and the proposed Theoretical Models. The researchers used multiple Fit Indices to comprehensively evaluate the Model Fit. These indices include the Chi-square to Degrees of Freedom Ratio, the Root Mean Square Error of Approximation, the Tucker-Lewis Index, and the Comparative Fit Index. The researchers presented the results in Table 10. All Fit Indices met the acceptable criteria. The Chi-square to Degrees of Freedom Ratio reached 1.668, which remains below the threshold of 3. The Root Mean Square Error of Approximation was 0.039, which is below 0.08. Furthermore, both the Tucker-Lewis Index and the Comparative Fit Index exceeded the threshold value of 0.9. The authors presented the path coefficients of the Confirmatory Factor Analysis Models after fitting in Figure 2. These results indicate that the Measurement Models demonstrate a high degree of fit with the Theoretical Frameworks. This performance suggests that the model specification is reasonable.

**Table 10:** Confirmatory factor analysis model fit indices.

Commonly used indices	CMIN/DF	RMSEA	NFI	IFI	TLI	CFI
Evaluation Criteria	<3	<0.08	>0.9	>0.9	>0.9	>0.9
Fit Results	1.668	0.039	0.958	0.983	0.979	0.983

###### 3.4.3.2.2 Convergent validity

The authors define Convergent Validity as the extent to which Items reflect their corresponding Variables in terms of Factor Loadings. The researchers evaluated this validity using the Average Variance Extracted and Composite Reliability. The Average Variance Extracted measures the variance captured by a construct to indicate how well it explains its Observed Variables. Meanwhile, Composite Reliability reflects the internal consistency between a construct and its Items. According to established standards in Confirmatory Factor Analysis, Convergent Validity is confirmed when Standardized Factor Loadings exceed 0.500, Average Variance Extracted Values exceed 0.500, and Composite Reliability Values exceed 0.700. The researchers presented the results of the convergent validity analysis in Table 11. All Average Variance Extracted Values remained above 0.5, with a range from 0.685 to 0.809. Furthermore, all Composite Reliability Values exceeded 0.7, with a range from 0.867 to 0.944. These results indicate that each of the Observed Variables adequately represents its corresponding Latent Variables and that the Measurement Models demonstrate strong Convergent Validity. Moreover, all Standardized Factor Loadings were greater than 0.77. This consistency further confirms the stability of the Measurement Models and provides a solid basis for subsequent SEM Analysis.

**Table 11:** Convergence validity.

Variable	Path	Factor	Estimate	AVE	CR
AC1	←	Appearance characteristics	0.873	0.747	0.922
AC2	←		0.875		
AC3	←		0.894		
AC4	←		0.814		
FC1	←	Functional characteristic	0.783	0.69	0.899
FC2	←		0.833		
FC3	←		0.804		
FC4	←		0.896		
SC1	←	Symbolic characteristic	0.902	0.809	0.944
SC2	←		0.869		
SC3	←		0.894		
SC4	←		0.935		
SV1	←	Sustainability values	0.802	0.685	0.867
SV2	←		0.856		
SV3	←		0.826		
EV1	←	Emotional values	0.836	0.758	0.904
EV2	←		0.896		
EV3	←		0.883		
PD1	←	Purchase decisions	0.77	0.726	0.913
PD2	←		0.834		
PD3	←		0.816		
PD4	←		0.98		

#### 3.4.4 Descriptive statistical analysis

The researchers conducted comprehensive Descriptive Statistical Analyses for each latent variable and its corresponding indicators, with the detailed results presented in Table 12. The final data set consistently maintained a valid sample size of 432 respondents, which provides a sufficient empirical basis for the subsequent structural model testing. All variables were measured using a 7-point Likert Scale, ranging from a minimum value of 1 to a maximum value of 7, to accurately capture the nuanced perceptions of Generation Z consumers.

**Table 12:** Correlation analysis.

Variable	AC	FC	SC	EV	SV	PD
AC	0.864					
FC	0.473**	0.831				
SC	0.212**	0.233**	0.899			
EV	0.58**	0.351**	0.181**	0.871		
SV	0.271**	0.305**	0.367**	0.256**	0.828	
PD	0.496**	0.398**	0.329**	0.49**	0.428**	0.852

** denotes *p* < 0.01, the diagonal values represent the square roots of AVE.

The mean scores for all measured variables were relatively high, ranging from 5.046 to 6.278, which indicates that the respondents generally held positive attitudes toward the assessed attributes of sustainable denim. Notably, the variables related to Purchase Decisions and Emotional Values exhibited the highest mean values, suggesting that consumers prioritize psychological satisfaction and final consumption intentions during the decision-making process. These figures imply that the aesthetic and affective appeal of Appearance Characteristics plays a pivotal role in shaping consumer preferences.

The standard deviations ranged from 0.761 to 1.307, reflecting moderate variability across the constructs and a reasonably distributed dataset. Furthermore, the researchers calculated the Skewness and Kurtosis for all items to assess the univariate normality of the data. The absolute values for Skewness were all below 2.0, and the values for Kurtosis were below 7.0, confirming that the data distribution did not significantly deviate from normality. These statistical properties ensure that the relationships between Functional Characteristics, Symbolic Characteristics, and Sustainability Values can be accurately estimated using Maximum Likelihood estimation. This robust descriptive foundation confirms the suitability of the data for higher-order multivariate analysis within the structural equation framework.

#### 3.4.5 Correlation analysis and discriminant validity

The researchers presented the results of the Correlation Analysis among the Latent Variables in Table 12. The values on the diagonal of the table represent the Square Roots of the Average Variance Extracted for each factor. These diagonal values exceed the Correlation Coefficients between each factor and other Factors. This pattern indicates that the Measurement Models demonstrate good Discriminant Validity. All pairwise Correlation Coefficients are statistically significant as the *p*-values are less than 0.01.

Specifically, Appearance Characteristics, Emotional Values, and Sustainability Values show relatively strong correlations with Purchase Decisions. These coefficients reached 0.496, 0.490, and 0.428, respectively. This result suggests that consumers place greater emphasis on Appearance Characteristics, Emotional Values, and Sustainability Values during their decision-making process. Additionally, Symbolic Characteristics and Functional Characteristics exhibit lower but still significant Correlation Coefficients with Purchase Decisions. These values were 0.329 and 0.398, respectively. These findings indicate that Symbolic Characteristics and Functional Characteristics also play a certain role in consumer decision-making. The Research Variables not only display significant positive correlations but also demonstrate good Discriminant Validity. This performance meets the applicability requirements for SEM Analysis.

Furthermore, the researchers calculated the Heterotrait-Monotrait Ratio of Correlations to address potential Conceptual Overlaps and provide a more conservative assessment of Discriminant Validity (Henseler et al., 2015). The authors presented the results in Table 13. The Heterotrait-Monotrait Value between Appearance Characteristics and Emotional Values was 0.712, which is well below the stringent 0.85 threshold. Additionally, all other Heterotrait-Monotrait Values in the matrix remained within acceptable limits. These findings confirm that despite their strong Conceptual Linkages, the two Constructs possess distinct Discriminant Validity.

**Table 13:** Discriminant validity: Heterotrait–Monotrait ratio (HTMT).

Variable	AC	FC	SC	EV	SV	PD
AC	–					
FC	0.412	–				
SC	0.355	0.521	–			
EV	0.712	0.432	0.398	–		
SV	0.488	0.288	0.312	0.545	–	
PD	0.622	0.344	0.387	0.599	0.678	–

#### 3.4.6 Structural equation model analysis

**Figure 3:**
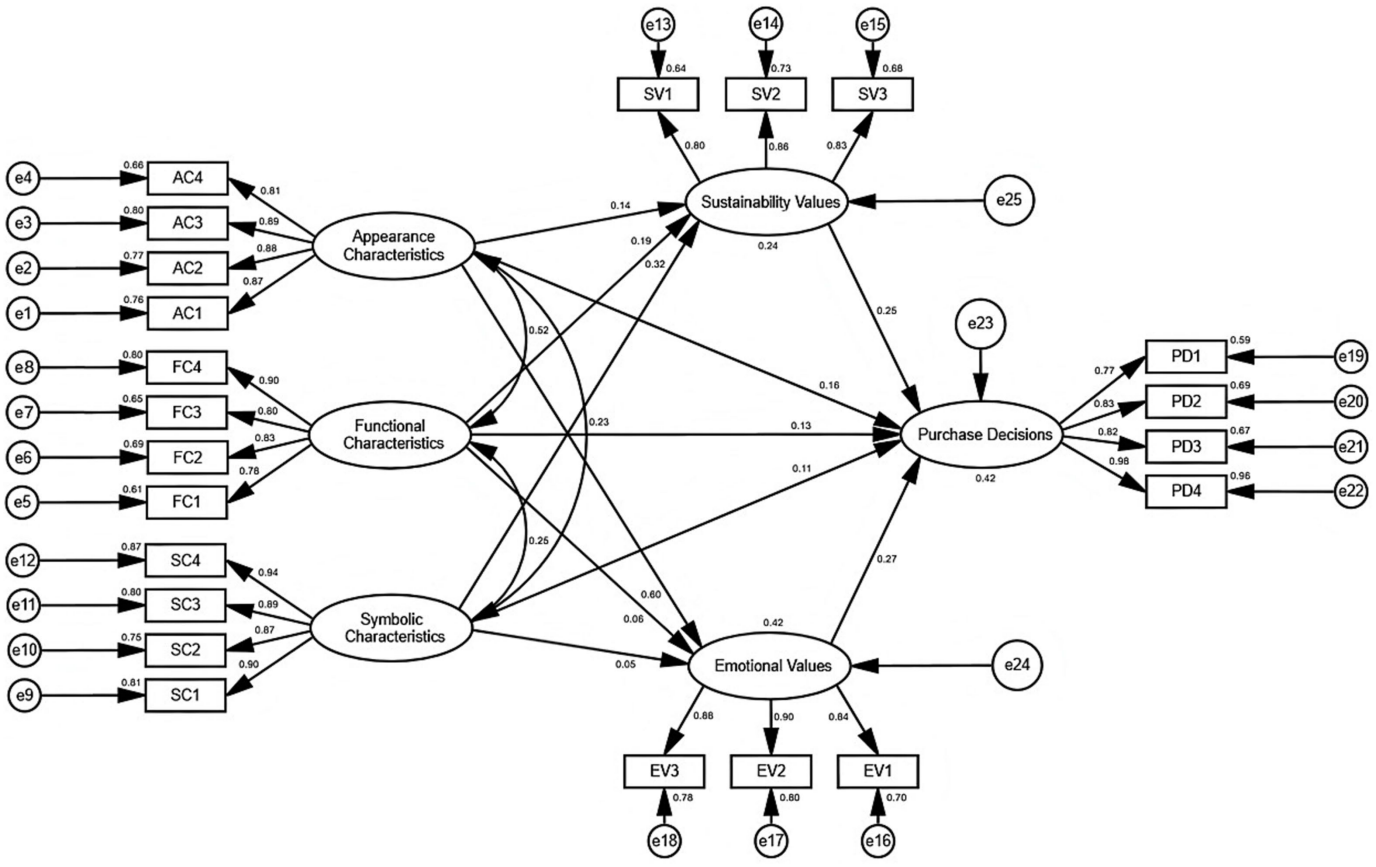
Structural equation model.

The Structural Equation Model serves as an analytical method for measuring Relationships between different Variables. The research data passed the Validity Tests and demonstrated good Reliability and Validity. Therefore, the authors employed AMOS 26 to construct the Structural Equation Models based on the Model Assumptions to validate the Relationships among Variables. The authors presented the visual representation of these results in Figure 3. The Structural Equation Models incorporate 6 Variables, 22 Observed Variables, and 25 Residual Variables.

##### 3.4.6.1 Model fit test

The researchers commonly use Model Fit to assess the degree of alignment between Hypotheses and Research Data. Based on the Model Fit Calculations performed by AMOS 26 software, the authors presented the Fit Indices corresponding to the Structural Equation Models in Table 14. The Structural Equation Models yielded a Chi-square to Degrees of Freedom Ratio of 1.668, which is less than 3. The Root Mean Square Error of Approximation reached 0.039, which remains below the threshold of 0.08. Furthermore, the Incremental Fit Index, the Normed Fit Index, and the Comparative Fit Index all exceeded 0.9. The Parsimony Goodness-of-fit Index and the Parsioned Normed Fit Index remained above 0.5. These results indicate a good overall Model Fit for the study.

**Table 14:** Model fit indices.

Index category	Evaluation index	Fit criteria	Model fit results	Whether standard is met
Absolute fit measures	CMIN/DF	<3.0	1.668	Yes
	GFI	>0.9	0.936	Yes
	AGFI	>0.9	0.917	Yes
	RMSEA	<0.08	0.039	Yes
Incremental fit index	IFI	>0.9	0.983	Yes
	NFI	>0.9	0.958	Yes
	CFI	>0.9	0.983	Yes
Parsimonious fit index	PGFI	>0.5	0.721	Yes
	PNFI	>0.5	0.808	Yes

##### 3.4.6.2 Specific path results

After establishing the Structural Equation Models and conducting Model Path Significance Tests using the software, the researchers obtained the Standardized Path Coefficients of Influencing Factors, the Critical Ratio Values, and the Significance Levels. Generally, if a Standardized Coefficient is greater than or equal to 0.1 and the Critical Ratio Value exceeds 1.96, the Path Coefficients pass the Significance Test at the 95% Confidence Level. Furthermore, the *p*-value must be less than 0.05. This result indicates that the corresponding Path Hypotheses in the proposed Structural Equation Models are supported. Otherwise, the researchers do not support the Path Hypotheses. The authors presented the results of the Path Analysis for the Research Models in Table 15.

**Table 15:** Structural equation model path coefficients.

Path	Unstandardized coefficient	Standardized coefficient	S.E.	C.R.	*p*
SV ← AC	0.105	0.145	0.043	2.475	0.013
EV ← AC	0.526	0.600	0.050	10.621	***
SV ← FC	0.179	0.189	0.057	3.151	0.002
EV ← FC	0.067	0.058	0.060	1.119	0.263
EV ← SC	0.040	0.049	0.036	1.118	0.263
SV ← SC	0.220	0.322	0.035	6.271	***
PD ← AC	0.104	0.164	0.039	2.682	0.007
PD ← FC	0.106	0.128	0.042	2.534	0.011
PD ← SC	0.067	0.112	0.027	2.513	0.012
PD ← EV	0.195	0.270	0.041	4.715	***
PD ← SV	0.215	0.246	0.044	4.899	***

* indicates *p* < 0.050, ** indicates *p* < 0.010, *** indicates *p* < 0.001.

###### 3.4.6.2.1 Appearance characteristics

Appearance Characteristics have a significant positive effect on Purchase Decisions. The Standardized Estimate was 0.164, and the Critical Ratio reached 2.682. The *p*-value was 0.007. These results indicate that Generation Z consumers consider the visual design of products as an important factor influencing Purchase Decisions when selecting sustainable denim clothing. High aesthetic appeal in style and overall design aesthetics can enhance Purchase Intentions. This alignment with Generation Z consumption habits emphasizes individuality and fashion sense. Therefore, the researchers support Hypothesis H11.

The relationship between Appearance Characteristics and Sustainability Values is significant. The Standardized Estimate was 0.145, and the Critical Ratio was 2.475. The *p*-value reached 0.013. This finding suggests that when Generation Z consumers evaluate the Sustainability Values of denim clothing, appearance design also plays a role. Fashionable sustainable apparel improves the perception of environmental attributes. These observations demonstrate that younger consumers accept the concept of green fashion. Consequently, the authors support Hypothesis H21.

The association between Appearance Characteristics and Emotional Values is the strongest among the measured paths. The Standardized Estimate reached 0.600, and the Critical Ratio was 10.621. The *p*-value was less than 0.001. This result indicates that the appearance of denim clothing is most strongly associated with the Emotional Values experienced by Generation Z consumers. Consumers do not only pursue visual aesthetics. They may develop stronger Purchase Intentions due to pleasure or a sense of belonging derived from the design. Thus, the researchers support Hypothesis H31.

###### 3.4.6.2.2 Functional characteristics

Functional Characteristics are significantly associated with Purchase Decisions. The Standardized Estimate was 0.128, and the Critical Ratio reached 2.534. The *p*-value was 0.011. These figures indicate that Generation Zconsumers pay attention not only to appearance but also to product functionality when choosing sustainable denim clothing. Specific features such as detachable designs, utility pockets, or versatility for multiple styling scenarios may increase Purchase Intentions. Therefore, the researchers support Hypothesis H12.

Functional Characteristics have a significant relationship with Sustainability Values. The Standardized Estimate was 0.189, and the Critical Ratio reached 3.151. The *p*-value was 0.002. This result indicates that Generation Z consumers may associate denim apparel possessing strong functionality with Sustainability Values. Durable apparel reduces replacement frequency, which aligns with environmental protection concepts. Consequently, the authors support Hypothesis H22.

In contrast, Functional Characteristics have no significant relationship with Emotional Values. The Standardized Estimate was 0.058, and the Critical Ratio was 1.119. The *p*-value reached 0.263, which exceeds the required significance threshold. This outcome indicates that Generation Z consumers do not derive stronger Emotional Values from purchasing denim apparel due to Functional Characteristics. Thus, the researchers do not support Hypothesis H32.

###### 3.4.6.2.3 Symbolic characteristics

Symbolic Characteristics are significantly associated with Purchase Decisions. The Standardized Estimate was 0.112, and the Critical Ratio reached 2.513. The *p*-value was 0.012. This result indicates that Generation Z consumers purchasing sustainable denim clothing are associated by Symbolic Characteristics, expressions of individuality, and Social Connections. Therefore, the researchers support Hypothesis H13.

Symbolic Characteristics have a significant relationship with Sustainability Values. The Standardized Estimate was 0.322, and the Critical Ratio reached 6.271. The *p*-value was less than 0.001. This finding indicates that Generation Z consumers may associate the Symbolic Characteristics of denim apparel with sustainability concepts. Consequently, the authors support Hypothesis H23.

In contrast, Symbolic Characteristics have no significant relationship with Emotional Values. The Standardized Estimate was 0.049, and the Critical Ratio was 1.118. The *p*-value reached 0.263, which exceeds the required significance threshold. These statistics indicate that for Generation Z consumers, the symbolic nature of products does not directly enhance Emotional Values when purchasing denim apparel. Thus, the researchers do not support Hypothesis H33.

###### 3.4.6.2.4 Sustainability values

Sustainability Values have a significant relationship with Purchase Decisions. The Standardized Estimate was 0.246, and the Critical Ratio reached 4.899. The *p*-value was less than 0.001. This result indicates that Generation Zconsumers are associated by the environmental characteristics of products during their decision-making process. As Environmental Awareness increases, consumers are more willing to choose denim clothing made from eco-friendly materials, produced through sustainable processes, or incorporating recycling. These findings demonstrate that sustainability factors directly shape consumer choices. Therefore, the researchers support Hypothesis H4.

###### 3.4.6.2.5 Emotional values

Emotional Values have a significant relationship with Purchase Decisions. The Standardized Estimate was 0.270, and the Critical Ratio reached 4.715. The *p*-value was less than 0.001. This result indicates that the Purchase Decisions of Generation Z consumers are associated with Emotional Values. These consumers tend to purchase denim clothing that provides satisfaction, pleasure, or social recognition. Consequently, these findings demonstrate that psychological and emotional factors are critical factors associated with consumer behavior. Therefore, the researchers support Hypothesis H5.

#### 3.4.7 Mediation effect analysis

To further scrutinize the internal mechanisms between product attributes and Purchase Decisions, the researchers utilized the Bootstrap Mediation Effect Analysis. The authors performed the sampling 5,000 times with a 95 percent Confidence Interval. The authors summarized the results of the specific Indirect Effects in Table 16.

**Table 16:** Mediation effect analysis.

Mediation	Effect	S.E.	Lower	Upper	*p*	Significance
AC → SV → PD	0.026	0.011	0.012	0.049	0.006	Partial mediation
AC → EV → PD	0.015	0.009	0.004	0.033	0.009	Partial mediation
FC → SV → PD	0.040	0.017	0.015	0.073	0.007	Partial mediation
FC → EV → PD	0.002	0.010	−0.012	0.020	0.654	No significant mediation
SC → SV → PD	0.049	0.025	0.017	0.096	0.007	Partial mediation
SC → EV → PD	0.016	0.015	−0.002	0.048	0.153	No significant mediation

Regarding the mediating role of Sustainability Values, the results indicate that all three indirect paths are statistically significant. Specifically, Sustainability Values mediate the relationship between Appearance Characteristics and Purchase Decisions in Hypothesis Ha. The Effect Size was 0.026, and the Probability Value reached 0.006. The 95 percent Confidence Interval ranged from 0.012 to 0.049. Furthermore, Sustainability Values mediate the relationship between Functional Characteristics and Purchase Decisions in Hypothesis Hb. The Effect Size was 0.040, and the Probability Value was 0.007. The 95 percent Confidence Interval ranged from 0.015 to 0.073. Finally, Sustainability Values mediate the relationship between Symbolic Characteristics and Purchase Decisions in Hypothesis Hc. The Effect Size reached 0.049, and the Probability Value reached 0.007. The 95 percent Confidence Interval ranged from 0.017 to 0.096. These findings suggest that Sustainability Values act as a foundational cognitive bridge that converts various product attributes into green consumption intentions.

Crucially, the results exhibit a distinct asymmetry regarding the mediating role of Emotional Values. As hypothesized in Hypothesis Hd, Appearance Characteristics exert a significant Indirect Effect on Purchase Decisions through Emotional Values. The Effect Size was 0.015, the Probability Value reached 0.009, and the 95% Confidence Interval ranged from 0.004 to 0.033. However, the indirect paths from Functional Characteristics and Symbolic Characteristics to Purchase Decisions through Emotional Values are strictly non-significant. For Hypothesis He, the indirect effect of Functional Characteristics on Purchase Decisions through Emotional Values was not statistically significant where *β* = 0.002, *p* = 0.654, and the 95% Confidence Interval ranged from −0.010 to 0.019. This indicates that Emotional Values do not play a mediating role in this relationship, and Hypothesis He is not supported. Similarly, for Hypothesis Hf, the indirect effect of Symbolic Characteristics on Purchase Decisions mediated by Emotional Values was also non-significant where β = 0.016, *p* = 0.153, and the 95% Confidence Interval ranged from −0.003 to 0.048. Consequently, no significant mediation was observed for Symbolic Characteristics through Emotional Values, and Hypothesis Hf is not supported.

Consequently, while Sustainability Values act as a universal mediator, the role of Emotional Values remains selective and primarily triggered by aesthetic appeal. This finding indicates a lack of significant emotional mediation for functional and symbolic variables in this context. Generation Z consumers do not experience significantly enhanced emotional resonance based solely on practical functionality or abstract brand symbolism. This phenomenon results in a strong visual priority effect. In this market, only superior aesthetic appearance successfully translates into the emotional gratification necessary to predict final Purchase Decisions for sustainable denim.

The Mediation Effect Analysis reveals that Sustainability Values partially mediate the relationship between Appearance Characteristics and Purchase Decisions. The researchers supported Hypothesis Ha. The Effect Size was 0.026, and the Probability Value reached 0.006. These findings indicate that when evaluating sustainable denim clothing, Generation Z consumers are not only directly associated by Appearance Characteristics in terms of Purchase Intentions, but also indirectly through enhanced environmental awareness which is further linked to consumption decisions. Therefore, brands should emphasize the use of eco-friendly materials and sustainable production techniques while innovating in appearance design to enhance the perception of sustainability among consumers.

Furthermore, Sustainability Values partially mediate the relationship between Functional Characteristics and Purchase Decisions. The researchers supported Hypothesis Hb. The Effect Size was 0.040, and the Probability Value reached 0.007. This result suggests that consumers may associate highly functional designs, such as durability and detachable designs, with Sustainability Values. Thus, designers can focus on garment durability and eco-friendly processes to reinforce this perception.

Additionally, Sustainability Values significantly and partially mediate the relationship between Symbolic Characteristics and Purchase Decisions. The researchers supported Hypothesis Hc. The Effect Size reached 0.049, and the Probability Value was 0.007. These statistics indicate that brand culture and environmental responsibility can strengthen the Sustainability Values held by consumers, thereby influencing Purchase Intentions. Therefore, brands can enhance the environmental awareness and sense of brand belonging of consumers through community marketing or environmental public welfare activities.

Regarding the mediating role of Emotional Values, Appearance Characteristics exert a partial mediating effect on Purchase Decisions through Emotional Values. The researchers supported Hypothesis Hd. The Effect Size was 0.015, and the Probability Value reached 0.009. These statistics indicate that when purchasing sustainable denim clothing, consumers are not only directly attracted by visual design, but also experience increased Purchase Intentions due to emotional pleasure derived from the products. Therefore, brands can enhance the emotional resonance of consumers by strengthening the individuality-oriented designs and fashion appeal of their products.

However, the relationships between Functional Characteristics and Purchase Decisions, as well as Symbolic Characteristics and Purchase Decisions, through Emotional Values lack statistical significance. The researchers did not support Hypothesis He or Hypothesis Hf because the Probability Values exceeded 0.05. These results indicate that consumers do not experience significant emotional responses when purchasing denim clothing solely due to Functional Characteristics or Symbolic Characteristics. Therefore, brands should focus more on how aesthetic design shapes the Emotional Values of consumers. The authors suggest that companies should not overly rely on functional attributes or symbolic meanings to enhance emotional identification in the sustainable denim market.

## 4 Results

### 4.1 Preliminary analysis and normality test

Before conducting the structural model analysis, data normality was assessed. The absolute values of skewness for all items ranged from 0.124 to 1.156, and kurtosis ranged from 0.054 to 1.872, both well within the recommended thresholds (skewness < 3, kurtosis < 10), confirming multivariate normality. Additionally, Harman’s single-factor test was performed to address Common Method Variance (CMV). The first extracted factor accounted for 36.4% of the total variance, below the 40% threshold, indicating that CMV does not significantly bias the study’s findings.

### 4.2 Measurement model and discriminant validity

The measurement model demonstrated excellent fit (CMIN/DF = 1.668, CFI = 0.983, NFI = 0.958, RMSEA = 0.039). As shown in Table 12, all factor loadings exceeded 0.70, and Average Variance Extracted (AVE) values were above 0.50, ensuring convergent validity.

To provide a more conservative assessment of discriminant validity, the Heterotrait-Monotrait (HTMT) ratio was calculated (see Table 13). All HTMT values were below the stringent 0.85 threshold (e.g., HTMT between AC and EV was 0.712), confirming that the constructs are empirically distinct despite their conceptual linkages.

### 4.3 Mediation effect analysis

The results of the bootstrap mediation analysis (5,000 samples) are presented in Table 16. Sustainability Value demonstrates a consistent mediating role across all three paths, with significant indirect effects observed for AC → SV → PD (Effect = 0.026, *p* = 0.006), FC → SV → PD (Effect = 0.040, *p* = 0.007), and SC → SV → PD (Effect = 0.049, *p* = 0.007), all indicating partial mediation.

Regarding Emotional Value, a significant partial mediation effect is observed only for the path AC → EV → PD (Effect = 0.015, *p* = 0.009). In contrast, the indirect effects for FC → EV → PD (Effect = 0.002, *p* = 0.654) and SC → EV → PD (Effect = 0.016, *p* = 0.153) are not significant, indicating no significant mediation for these paths.

These findings suggest that Emotional Value mediates the relationship between Appearance Characteristics and Purchase Decision, whereas no significant mediation effects are observed for Functional or Symbolic Characteristics through Emotional Value.

## 5 Discussion

### 5.1 The dual-pathway mechanism of sustainable consumption

This study examines the Psychological Mechanisms underlying Generation Z Consumers regarding Purchase Decisions of sustainable denim clothing by integrating the S-O-R Frameworks, VBN Theories, and Kansei Engineering. The findings reveal a clear pattern in which different Product Attributes are associated with Consumer Responses through both Cognitive Pathways and Affective Pathways. The researchers believe these results contribute to the literature by demonstrating how Product Design Attributes related to Behavioral Intentions.

### 5.2 Visual priority and the dominance of aesthetic stimuli

The results highlight the particularly prominent role of Appearance Characteristics, which are positively associated with Purchase Decisions as well as with both Sustainability Values and Emotional Values. The researchers observed a notably high Path Coefficient of 0.600 from Appearance Characteristics to Emotional Values. The authors argue that this Disproportionate Dominance reflects a Visual Priority pattern among Generation Z Consumers, where Design Elements act as salient External Stimuli that rapidly activate Affective Reactions.

To address potential concerns regarding Measurement Artefacts, the researchers performed Discriminant Validity Tests using the Heterotrait-Monotrait Ratio. The results indicated that while Appearance Characteristics and Emotional Values share some Conceptual Proximity due to Hedonic Aesthetics, they remain distinct psychological constructs. The authors confirmed that Appearance Characteristics focus on Tangible Textures, whereas Emotional Values capture Intangible psychological satisfactions. This strong relationship suggests that Aesthetic Impressionsserve as the primary trigger for Emotional Resonances in the context of Sustainable Fashion.

### 5.3 The centrality of sustainability values as a cognitive mediator

Beyond the role of Visual Designs, the findings underscore the central importance of Sustainability Values as a Psychological Mechanism linking Product Attributes to Behavioral Intentions. Rather than directly shaping Purchase Decisions, Design-Related Characteristics appear to be interpreted through the lens of Perceived Sustainability. This pattern is consistent with the Value-Belief-Norm Theories, which emphasize that Pro-environmental Behaviors are associated with Internalized Values. In this study, Sustainability Values captures not only Functional Aspects such as Durability, but also broader environmental responsibilities.

### 5.4 Selective emotional engagement and functional baseline expectations

In contrast, the role of Emotional Values appears to be more selective. Although Emotional Values is positively associated with Purchase Decisions, its Mediating Effect is primarily observed in relation to Appearance Characteristics. The researchers found no significant mediation for Functional Characteristics or Symbolic Characteristics. This indicates that Emotional Engagement is closely tied to Aesthetic Experiences rather than to Utilitarian Aspects. Furthermore, the weak associations of Functional Characteristics suggest that Generation Z Consumers perceive these as Baseline Expectations rather than differentiating factors. The authors also note that Symbolic Characteristics related to Social Signaling do not strongly resonate with this Participant Group.

### 5.5 Methodological limitations and future research directions

Several limitations should be acknowledged. The researchers emphasize that the cross-sectional nature of the Data Points restricts the ability to infer Causal Relationships. Because the authors collected all Information from a single Survey Questionnaire at one specific time point, the risk of Common Method Variance cannot be entirely eliminated. While the researchers performed Harman’s Single-Factor Test to mitigate these concerns, the authors recommend that future Studies should utilize Longitudinal Designs or multiple Information Sources to further minimize the potential for Common Method Bias.

The current study focuses exclusively on Chinese Generation Z consumers, with a significant proportion of the sample consisting of university students. While this demographic represents a key driver of the sustainable fashion market, such a specific sample composition may limit the generalisability of the findings to global populations or older age cohorts. Cultural nuances and varying socio-economic conditions in different regions could potentially moderate the relationships between Appearance Characteristics, Emotional Values, and Purchase Decisions. Consequently, the results should be interpreted with caution when applied to broader international contexts. We encourage future researchers to conduct cross-cultural comparative studies to validate the universal applicability of our model. Furthermore, subsequent academic investigations should adopt more stringent validity assessments to examine potential conceptual overlaps between aesthetic constructs and emotional constructs across diverse market environments. This would refine the theoretical boundaries of Sustainability Values and provide a more nuanced understanding of consumer behavior in the global denim industry.

### 5.6 Practical implications derived from empirical evidences

The researchers derived several Practical Implications for Apparel Designers and Industry Stakeholders based on the specific Path Coefficients and mediation effects identified in the structural model. Because Appearance Characteristics exhibited the strongest Direct Effect on Emotional Values at 0.600, which in turn serves as the primary driver for the significant Mediation Path leading to Purchase Decisions, the authors recommend that Sustainable Denim Products should prioritize Aesthetic Innovations. This strategy is essential to trigger the immediate Affective Responses that bridge visual stimuli and consumer behavior. The empirical dominance of this path suggests that the aesthetic appeal of denim is the primary vehicle through which abstract sustainability is translated into tangible consumer pleasure, making visual design the most effective driver for elevating final Purchase Decisions within the emotional dimension.

In contrast, the empirical findings revealed that Emotional Values do not function as a significant mediator for Functional Characteristics or Symbolic Characteristics. Instead, the influence of these two attributes on Purchase Decisions is primarily channeled through the mediation of Sustainability Values. Consequently, the authors argue that design strategies for functional and symbolic dimensions must be systematically linked to perceived environmental ethics rather than emotional appeal. To reinforce the structural path from the structural path from Functional Characteristics to Purchase Decisions, the researchers propose focusing on design solutions that emphasize product longevity and resource efficiency. Such attributes provide objective evidence of durability, which the data suggest are essential for strengthening the consumer’s belief in the environmental benefits of the garment, thereby activating the moral obligations defined in the Value-Belief-Norm Theories.

The authors suggest that Marketing Communications should focus on the Symbolic Characteristics that align with the environmental responsibilities of Generation Z. Since the symbolic impact on Purchase Decisions relies on the mediation of Sustainability Values rather than Emotional Values, stakeholders should prioritize transparency and ethical storytelling. By enhancing the “communicative value” of sustainable denim, designers can ensure that Symbolic Characteristics resonate with the self-identity and ethical commitments of young consumers. By grounding these strategic recommendations in the specific significant and non-significant paths of the Quantitative Results, the researchers ensure that the Practical Applications are directly supported by the Empirical Data, providing a scientifically valid roadmap for the development of sustainable apparel.

## 6 Conclusion

This study suggests that Sustainability Value is consistently associated with the relationship between product attributes and purchase intention, whereas Emotional Value plays a more selective role, primarily associated with visual aesthetics. The findings indicate that Generation Z consumers’ Purchase Decisions is most strongly associated with aesthetic appeal and perceived Sustainability Values.

From a practical perspective, fashion enterprises may consider adopting a “visual-centric sustainability” strategy. Rather than emphasizing complex technical functions that may be less likely to resonate emotionally, designers may place greater emphasis on expressive sustainable elements, such as unique eco-washes or architectural silhouettes, that are associated with emotional responses. Marketing communication may also benefit from shifting from abstract brand symbolism to more concrete expressions of sustainability, in order to better align green awareness with purchase behavior.

This study utilized a convenience sample of Chinese Generation Z consumers, which may limit the generalizability of the results to other cultural or age cohorts. Furthermore, the cross-sectional design reflects associations rather than causality. Future research could employ longitudinal designs or experimental methods to further examine these psychological mechanisms across diverse contexts.

## Data Availability

The datasets presented in this study can be found in online repositories. The names of the repository/repositories and accession number(s) can be found in the article/supplementary material.
